# Molecular Detection of *Toxoplasma gondii* in Enclosures of African Primates (*Papio papio* and *Papio hamadryas*) at the Municipal Zoo of Bauru, São Paulo State, Brazil

**DOI:** 10.1111/jmp.70077

**Published:** 2026-04-24

**Authors:** Emilly Ribeiro, Michelle Santos Sabioni, Michel dos Santos Pinto, João Alfredo Biagi Camargo Neto, Wesley José dos Santos, Lívia Maísa Guiraldi, Isabella Neves Aires, Katia Denise Saraiva Bresciani, Antônio Carlos Paes, Simone Baldini Lucheis

**Affiliations:** ^1^ Faculdade de Medicina Veterinária e Zootecnia (FMVZ) Universidade Estadual Paulista (Unesp) São Paulo Brazil; ^2^ Faculdade de Medicina Veterinária (FMV) Universidade Estadual Paulista (Unesp) São Paulo Brazil; ^3^ Faculdade de Medicina de Botucatu (FMB) Universidade Estadual Paulista (Unesp) São Paulo Brazil

**Keywords:** diagnosis, oocysts, toxoplasmosis, zoonosis

## Abstract

Toxoplasmosis is one of the most prevalent parasitic infections in animals and humans worldwide, attracting the attention of many researchers who, in recent decades, have identified the sources of *Toxoplasma gondii* infections to optimize the adoption of preventive measures. In previous studies, it has been found that humans are infected mainly by consuming raw or undercooked meat or by ingesting fruits, vegetables, seafood or water contaminated with oocysts of this protozoan. Soil contaminated with *T. gondii* oocysts is a source of infection for animals and humans, but it has rarely been directly detected due to the lack of appropriate methods. Given that toxoplasmosis is a widespread zoonosis of great public health importance, we investigated the occurrence of *T. gondii* in the soil of 30 enclosures housing different animal species at the Bauru Zoo, São Paulo, by amplifying the protozoan's DNA using conventional polymerase chain reaction (cPCR). The cPCR for *T. gondii* was performed using primers TOX4 and TOX5, which amplify 529 bp. Thus, we observed that soil samples from two enclosures of African primates of the species *Papio papio* (Guinea Baboon) and *Papio hamadryas* (Hamadryas Baboon) were positive by PCR for *T. gondii*, an unprecedented result in the literature.

## 1 Introduction

Toxoplasmosis is a parasitic infection widely distributed worldwide, caused by the obligate intracellular protozoan *Toxoplasma gondii*, an agent of public health relevance that can infect various animals, including humans [1]. Toxoplasmosis is classified as a zoonosis and represents an emerging Public Health concern, being associated with high morbidity and mortality rates, particularly in developing countries. It is estimated that approximately one third of the world's population has already been exposed to the etiological agent of the disease [2].

Felids are the only definitive hosts and are responsible for releasing oocysts into the environment [3, 4, 5]. However, other mammals, such as non‐human primates, can act as intermediate hosts [6]. Transmission of this protozoan can occur through several routes, the most common being the ingestion of food or water contaminated with sporulated oocysts and the consumption of raw or undercooked meat containing tissue cysts [7]. The potential for environmental contamination is worrying, as a single feline can excrete 1 billion oocysts in its feces, which are highly resistant to environmental conditions and can remain viable in the soil for months [8].

Infections caused by *T. gondii* have been described predominantly in non‐human primates kept in captivity [9, 10], in addition to some reports of outbreaks in marmosets in wild environments. New World primate species are considered highly susceptible to the acute and frequently fatal form of toxoplasmosis, and may present severe hepatic lesions, interstitial pneumonia, and systemic involvement affecting multiple organs [9, 10, 11]. In humans, severe manifestations of the disease are usually associated with immunosuppression, resulting in systemic alterations such as encephalitis, pneumonia, and hepatitis [12, 13, 14]. However, to date, these associations have not been observed in non‐human primates [15].

Studies indicate that the seroprevalence of *T. gondii* in non‐human primates is significantly lower in free‐ranging individuals (11.6%) when compared to those kept in captivity (59.6%) [16]. In this context, infection becomes more relevant in captive environments, since several species may develop severe forms of toxoplasmosis, often culminating in death [17].

Furthermore, non‐human primates kept in captivity may act as important reservoirs of *T. gondii*, also functioning as sentinels in the detection of possible sources of contamination in environments such as zoos. The role of wild animals as reservoirs of zoonoses, both in natural environments and under captive conditions, has raised increasing concern, especially due to the risk of transmission of these diseases [18].

Zoos are environments that are particularly sensitive to the spread of this parasite, as animals kept in captivity are subject to more stress and are therefore susceptible to developing clinical symptoms of toxoplasmosis. It is worth noting that the presence of felines in these settings, combined with handling and cleaning practices that can spread oocysts, favors transmission between animals and poses risks to humans who work or visit the zoo [19].

Studies conducted in Brazilian zoos reported a high prevalence of *T. gondii* in mammals [20], birds, and reptiles [21], indicating exposure of these animals to the parasite and the need to understand its transmission. Therefore, the objective of this study was to investigate the occurrence of *T. gondii* in soil samples from animal enclosures at the Bauru Municipal Zoo, São Paulo state, Brazil.

## 2 Methods

### 2.1 Study Area and Population

**FIGURE 1:**
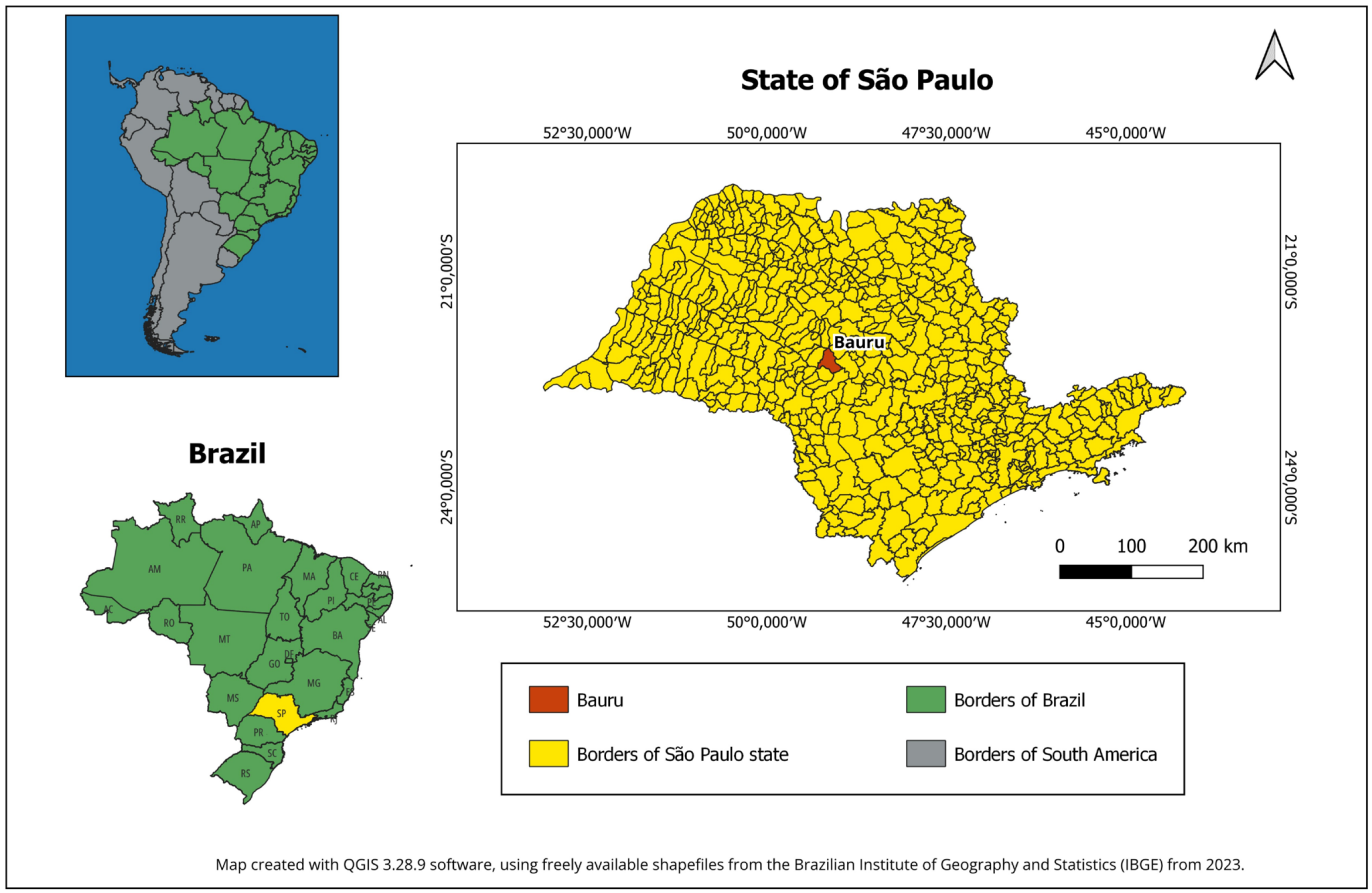
Maps of the state of São Paulo and Brazil, highlighting the municipality of Bauru.

This research was conducted at the Bauru Municipal Zoological Park, located in the western region of São Paulo state, Brazil (Figure 1). This is considered a very prominent zoo in the country, housing approximately 700 animals from 170 different species, including birds, reptiles, fish, and mammals, with an average of 210 000 visitors per year.

**FIGURE 2:**
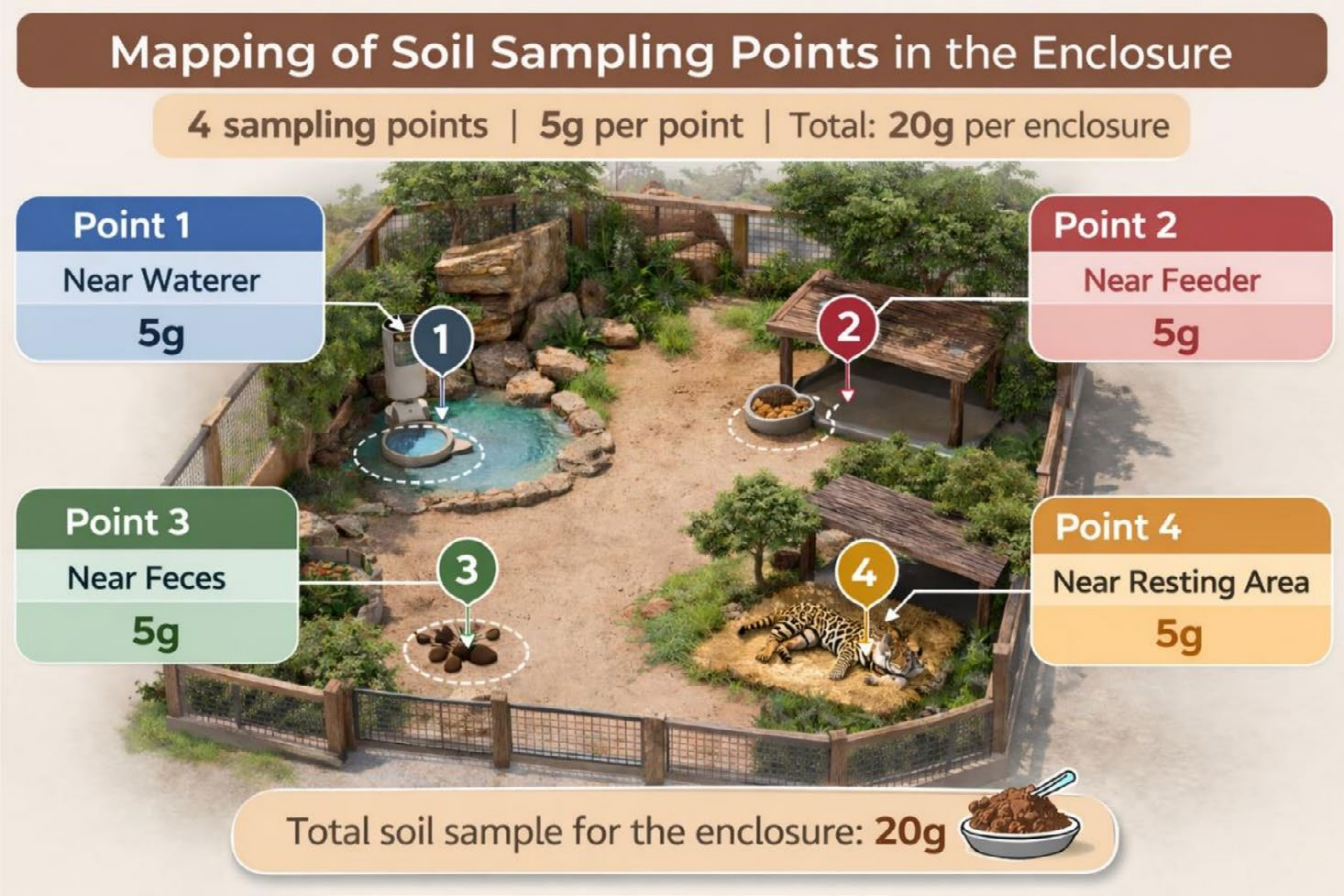
Illustration of the sampling points for soil collection within the animal enclosures. This image was created with the assistance of artificial intelligence (ChatGPT) for purely illustrative purposes.

Soil samples from 30 enclosures (Table 1) were collected and identified from 1 to 30. The collection points were divided into four categories, established according to the proximity to the animals' water (point 1), food (point 2), feces (point 3), and resting place (point 4), for a total of 120 samples. In the case of animals that did not rest in contact with the soil, the point was changed to the center of the enclosure (Figure 2).

**TABLE 1:** List of species with the number of enclosures investigated for the presence of *Toxoplasma gondii* at the municipal zoo of Bauru, state of São Paulo, Brazil.

Animal group/species	Scientific name
New World primates – 11 enclosures	
White‐whiskered spider monkey	*Ateles marginatus*
Brown howler monkey	*Alouatta fusca*
Red‐faced black spider monkey	*Ateles paniscus*
Black‐and‐gold howler monkey	*Alouatta caraya*
Red‐handed howler monkey	*Alouatta belzebul*
Common woolly monkey	*Lagothrix lagothricha*
Golden lion tamarin	*Leontopithecus rosalia*
Golden‐headed lion tamarin	*Leontopithecus chrysomelas*
Atlantic titi	*Callicebus personatus*
Saddleback tamarin	*Saguinus fuscicollis*
Red‐chested mustached tamarin	*Saguinus labiatus*
Peafowl – 5 enclosures	
Black‐shouldered peafowl	*Pavo cristatus* *mutation nigripennis*
Blue Indian peafowl	*Pavo cristatus*
White peafowl	*Pavo cristatus* *mutation alba*
Pied peafowl	*Pavo cristatus* *mutation pied*
Green peafowl	*Pavo muticus*
Water birds – 3 enclosures	
African sacred ibis	*Threskiornis aethiopicus*
Coscoroba swan	*Coscoroba coscoroba*
Whistling heron	*Syrigma sibilatrix*
Buff‐necked ibis	*Theristicus caudatus*
Scarlet ibis	*Eudocimus ruber*
Marsupial – 1 enclosure	
Red kangaroo	*Macropus rufus*
Felids – 6 enclosures	
Jaguarundi	*Puma yagouaroundi*
Ocelot	*Leopardus pardalis*
Puma	*Puma concolor*
Bengal tiger	*Panthera tigris tigris*
Lion	*Panthera leo*
Jaguar	*Panthera onca*
Old World primates – 4 enclosures	
Hamadryas baboon	*Papio hamadryas*
Mandrill	*Mandrillus sphinx*
Patas monkey	*Erythrocebus patas*
Guinea baboon	*Papio papio*

### 2.2 Sample Collection and Processing

The samples were collected from the superficial layer of the soil (at a depth of 5 cm) using previously sterilized plastic and stainless‐steel spoons, and were subsequently stored in sterilized 50 mL Falcon tubes to prevent contamination. Soil samples weighing 5 g, were collected from four points in each enclosure, then packaged in 50 mL plastic tubes and stored at −20°C.

*T. gondii* oocysts were recovered by centrifugal flotation in a hypersaturated sucrose solution. The collected samples were diluted by adding 10 mL of autoclaved deionized water for 15 min, and then vortexed for 1 min. Then, the resulting soil mix was overlaid with 20 mL of a cold (4°C) sugar solution (500 g dextrose and 6.5 g phenol crystals in 320 mL deionized water, s.g. = 1.2) and centrifuged at 1800 rcf for 20 min at 4°C. The supernatant of the mix was removed, and 35 mL of distilled water was added. The mixture was centrifuged again at 1800 rcf for 20 min at 4°C. The supernatant was removed and the pellet was resuspended in 200 μL of distilled water and subjected to genetic material extraction [22].

Genomic DNA extraction was performed with three different extraction kits, depending on laboratory availability. The commercial Kasvi Mini Spin Extraction Kit, the Cellco DNA Extraction Kit, and the Norgen Biotek Corp. Blood DNA Isolation Mini Kit were used, following each manufacturer's recommendations. The DNA extracted from the samples was quantified and stored at −20°C.

### 2.3 Molecular Detection of *Toxoplasma gondii*

Molecular detection of *T. gondii* was performed using the Polymerase Chain Reaction (PCR), with amplification of the 529‐bp non‐coding fragment and the use of primers TOX4 and TOX5 [23]. PCR was performed with 11 μL of PCR mix (50 mM KCl; 20 mM Tris–HCl; 1.6 mM MgCl2; 0.2 mM dNTPs; 1 U of Taq polymerase [Platinum Taq DNA Polymerase, Invitrogen]; 0.2 μM of each primer; and 8.3 μL of ultrapure water) and 1 μL of the sample. The reactions were subjected to initial denaturation at 94°C for 7 min and then to 35 cycles including denaturation (94°C for 1 min), annealing (55°C for 1 min), and extension (72°C for 1 min), with a final extension at 72°C for 10 min.

## 3 Results

In this study, of the 120 soil samples investigated, two tested positive for *T. gondii* using cPCR: one from the center of the Guinea baboon's ( *Papio papio* ) enclosure and the other from the area near the hamadryas baboon's ( *Papio hamadryas* ) food source.

## 4 Discussion

This study aimed to investigate the occurrence of *T. gondii* in soil samples from animal enclosures at the Bauru Municipal Zoo, São Paulo state, Brazil. Among the analyzed samples, two tested positive, both from African primate enclosures.

In Brazil, studies describing the molecular detection of *T. gondii* in soil samples are still scarce. However, in the state of Pernambuco, through PCR, oocysts of the aforementioned protozoan were detected in 8.33% (10/120) of soil samples collected in public places, such as squares, parks, universities, and hospitals in the city of Recife [24] and in 4.21% (4/95) of soil samples from 14 neighborhoods and four squares on Fernando de Noronha Island [25]. To date, there are no reports of the detection of *T. gondii* in soil samples from zoo enclosures in the country, making it impossible to make comparisons.

Soil contaminated with *T. gondii* oocysts may represent a risk factor for infections in zoo animals. This study lacks information on the origin of the sand used in the animal enclosures, which could have already been contaminated before being placed in these enclosures. However, it is known that the sand used was not autoclaved, which may have facilitated contamination and the spread of the parasite.

It is important to note that in 2013, several cases of toxoplasmosis were recorded at the Bauru Municipal Zoo, resulting in the deaths of several animal species, notably non‐human primates and 34 penguins, which presented *T. gondii* cysts in their brain tissue, identified through histopathology (unpublished data).

Although felids are the only definitive hosts capable of shedding *T. gondii* oocysts in their feces, in our study we did not detect DNA of this protozoan in the soil samples collected from the enclosures of these animals. However, *T. gondii* was identified in the enclosures of the African primates.

The African primates, Guinea baboon and hamadryas baboon, housed in the enclosures investigated in this study, were fed fruits, vegetables, feed (which was stored in bulk) and raw meat. The positive sample corresponding to the site near the *hamadryas* baboon's food may be due to environmental contamination through vegetables and fruits containing *T. gondii* oocysts, as these foods were fed to the animals directly on the ground. Various vegetables, such as lettuce, arugula, chicory, and parsley, have already been shown to transmit this parasite [26, 27].

In this research, the enclosures investigated were composed of concrete walls on the sides, a glass wall at the front, and the upper part was sealed with a grid. One of the possibilities for contamination of the places where primates were present would be the presence of domestic cats, which could have access to the enclosures and contaminate the soil by eliminating oocysts in their feces [28]. However, the complete sealing of the enclosures prevents the entry of felines and environmental contamination.

Despite the prior contamination of the sand used in the enclosures and the provision of vegetables directly on the ground being possible sources of environmental contamination, these factors were also associated with other enclosures in the zoo, including those of the felids, in which *T. gondii* DNA was not detected.

Therefore, the detection of *T. gondii* in zoos enclosures highlights a potential risk to the animals and even to humans who visit or work there, as the mode of environmental contamination is often uncertain. Therefore, possible preventive measures to be adopted would be the autoclaving of the enclosures' sand and avoiding contact of food with the soil.

## Funding

This work was supported by Fundação de Amparo à Pesquisa do Estado de São Paulo (Grant 2018/21617‐0).

## Ethics Statement

This study was approved by the Institutional Animal Care and Use Committee of Universidade Estadual Paulista—Câmpus de Botucatu/Faculdade de Medicina, under protocol number [1104/2014].

## Conflicts of Interest

The authors declare no conflicts of interest.

## Data Availability

Data sharing not applicable to this article as no data sets were generated analyzed during the current study.
